# An algebraic formula, deep learning and a novel SEIR-type model for the COVID-19 pandemic

**DOI:** 10.1098/rsos.230858

**Published:** 2023-08-02

**Authors:** A. S. Fokas, N. Dikaios, Y. C. Yortsos

**Affiliations:** ^1^ Department of Applied Mathematics and Theoretical Physics, University of Cambridge, Cambridge CB3 0WA, UK; ^2^ Mathematics Research Centre, Academy of Athens, 11527 Athens, Greece; ^3^ Viterbi School of Engineering, University of Southern California, Los Angeles, CA 90089, USA

**Keywords:** COVID-19 pandemic, SEIR model, deep learning, forecasting and mechanistic models

## Abstract

The most extensively used mathematical models in epidemiology are the susceptible-exposed-infectious-recovered (SEIR) type models with constant coefficients. For the first wave of the COVID-19 epidemic, such models predict that at large times equilibrium is reached *exponentially*. However, epidemiological data from Europe suggest that this approach is *algebraic*. Indeed, accurate long-term predictions have been obtained via a forecasting model only if it uses an algebraic as opposed to the standard exponential formula. In this work, by allowing those parameters of the SEIR model that reflect behavioural aspects (e.g. spatial distancing) to vary nonlinearly with the extent of the epidemic, we construct a model which exhibits asymptoticly *algebraic* behaviour. Interestingly, the emerging power law is consistent with the typical dynamics observed in various social settings. In addition, using reliable epidemiological data, we solve in a numerically robust way the *inverse problem* of determining all model parameters characterizing our novel model. Finally, using deep learning, we demonstrate that the algebraic forecasting model used earlier is optimal.

## 1. Introduction

In the framework of Holmdahl & Buckee [1], epidemiological models are broadly divided into two categories: *forecasting* and *mechanistic*. The former fit early-time data with a specific empirical formula, which is then used to predict the time evolution. The limitation of the forecasting models is that they usually remain valid for only a specific, short, period of time, during which the epidemiological situation is unchanged. For example, if a forecasting model is valid during part of a lockdown period, this model is not expected to make accurate predictions after the lockdown is lifted. As noted in Zhou *et al.* [2], forecasting models are ‘not well suited for long-term predictions’. Mechanistic models based on differential equations circumvent this limitation and can be used to make predictions even when the relevant circumstances change. Their limitation is that it is difficult to solve the associated *inverse problem*, namely, to determine the relatively large number of parameters involved in these models from the knowledge of epidemiological data.

**Figure 1.**
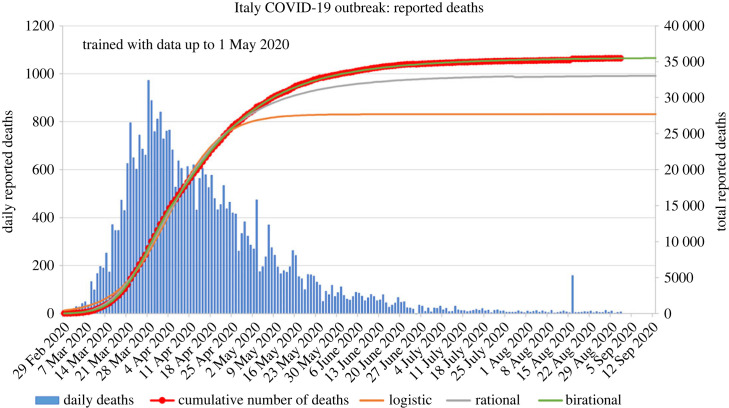
Actual data for the number of reported COVID-19 deaths for Italy (in blue and red) throughout the ‘first wave’ and predicted results using data until 1 May 2020. The birational formula (in green) is the only one among the three forecasting models (logistic, rational and birational) that matches well the actual data.

The first period of the COVID-19 pandemic, known as ‘the first wave’, was characterized by the fact that a single viral strain dominated the pandemic. This setting provides an ideal situation for the retrospective study of the various mathematical formulations (both forecasting and mechanistic) used in viral epidemics models. Based on this first wave, we previously introduced [3,4] a novel class of *forecasting* models, which provided accurate long-term predictions for the number of deaths in several European countries caused by the epidemic (figure 1). This was accomplished by using a simple algebraic formula as opposed to the standard exponential formula (logistic model). It is important to emphasize that the logistic formula, as well as similar formulae (see for example [5]) provide accurate fitting for the data of a *fixed* period. In particular, all these formulae can be used to model the data up to 1 May 2020. However, after fixing the parameters of these formulae using the data of a fixed period, these models failed to provide accurate predictions. On the other hand, after ‘training’ the algebraic formula of for the same period (i.e. after fixing the relevant parameters using data until 1 May 2020), this formula did provide accurate predictions for a period extending longer than 3.5 months, namely until 1 September 2020.

We show typical comparisons in figure 1, where we plot data for deaths during the first wave in Italy, as well as the predictions associated with three models, the logistic and the two novel models of [3,4]. The model routinely used in epidemiology is the logistic defined in (1.1) below; the rational and the birational formulae are given, respectively, by equations (1.2) and (1.3) below:

1.1 $$\;D(t)=\frac{D_{F}}{1+\beta e^{-\gamma t}},$$

1.2 $$D(t)=\frac{D_{F}}{1+\beta {\left(1+\delta t\right)}^{-\gamma }}$$

1.3 $$D(t)=\{\begin{array}{cc} \frac{c}{1+b{\left(1+dt\right)}^{-k}}, & t\leq T\\ \frac{c}{1+b{\left(1+dX\right)}^{-k}}-\frac{c_{1}}{1+b_{1}{\left(1+d_{1}X\right)}^{-k_{1}}}+\frac{c_{1}}{1+b_{1}{\left(1+d_{1}t\right)}^{-k_{1}}}, & t>T \end{array},$$

where *X* is a fixed parameter *X* usually taken to be *T*.

Here, *D*(*t*) denotes the cumulative number of deaths at time *t*, with the constant parameters *D_F_*, *β*, *γ* and *δ* determined by ‘training’ (fitting) the model with the data. Similar considerations are valid for the parameters defining the birational formula (1.3), where the time *T* corresponds to the inflection point of *D*(*t*) (details are given in [3,4]). Equation (1.1) shows that, for large *t*, the expression *D*(*t*) approaches its final value *D_F_* exponentially. In the case of the rational and birational models, the approach is algebraic. In this sense, it is better to refer to the rational formula as *algebraic* [3,4]. All three expressions in (1.1–1.3) are particular solutions of a specific differential equation (ODE) of the Riccati type, uniquely specified by the constant *D_F_* and by a function *a*(*t*). The formulae in (1.2) and (1.3) correspond to the case where *a*(*t*) is a rational function of *t*, whereas the logistic model corresponds to the case that *a*(*t*) is a constant. The logistic, rational and birational curves are shown along with real data in figure 1 above.

As shown in figure 1, the birational formula closely matches the actual data throughout the time interval considered, in contrast with the other two curves (rational and logistic). It is interesting that even though ‘training’ occurred during the lockdown period, its predictions remained accurate after the easing of the lockdown conditions. Possible explanations for this unexpected agreement are discussed in [6].

The fact that the correct asymptotic decay is algebraic rather than exponential suggests a social behaviour origin. Indeed, while most, although not all, physical and biological phenomena exhibit exponential decay in the distribution of key variables, or in the behaviour of correlation functions, this is not the case for social phenomena, where one usually observes algebraic dependences and power laws, perhaps because of self-similarity or fractal geometry notions [7]. Indeed, examples of power laws in social settings are ubiquitous, from economics to the distribution of income or of the populations of cities. While such behaviour may also be observed in natural phenomena, e.g. in long-range correlations, or near percolation thresholds in phase transitions [8], the key point regarding epidemiological models is the following: because epidemiological quantities are also affected by social behaviour, e.g. the relaxation of precautions as the epidemic wanes, it is likely that the apparent success of the birational equation is due to such phenomena. Using the *mechanistic* susceptible-exposed-infectious-recovered (SEIR) model and previously reported results in [9], we will demonstrate this assertion quantitatively in the following section.

The most extensively used mechanistic models in epidemiology are variations of the classical SIR model. This is based on the assumption of a constant total population, consisting of three subpopulations, namely, *susceptible*, *infected* and *recovered*, and three implicit conditions, that the control volume where the epidemic occurs is fixed, there is no influx of additional populations, and spatial gradients do not exist. Following chemical reaction engineering process analogies, Ramaswamy *et al.* [10] generalized the SIR model to a spatio-temporal model which relaxed all these restrictions; this led to a set of partial differential equations in terms of population densities. This generalization predicted the onset of spatio-temporal waves, as well as other characteristics inherently absent in the SIR model. In addition, it led to the emergence of a single dimensionless number that characterizes the process, the so-called reproduction number *R*_0_, quantitatively expressed as the ratio of the kinetics of infection to those of recovery [10]. Importantly, *R*_0_ incorporates a combination of physiological and biological parameters, and also reflects measures of social dynamics (e.g. via facial coverings, spatial distancing, areal density, etc.).

If *R*_0_ is constant, SIR predicts that any infection wave wanes asymptotically following an exponential behaviour. In previous work [9], we examined the question of what modifications of the model would lead to an algebraic rather than an exponential asymptotic behaviour, as appears to be the actual case in the first COVID-19 wave. Given that the only relevant parameter in SIR models is *R*_0_, we showed that for an algebraic behaviour to be obtained, it is necessary that beyond a certain time, *R*_0_ must become an increasing function of the infected fraction denoted by *i* (ratio of the number of infected individuals to the total). The particular dependence was found in [9] to be

1.4 $$R_{0}(i)\propto {\left(1+a\;i^{1/n}\right)}^{-1},$$

where the parameter *a* is a positive constant, and the exponent *n* was found to satisfy *n* > 2. Under these conditions, the asymptotic decay is algebraic and follows a power law in time with an exponent equal to − *n*/(*n* − 1). Equation (1.4) shows that in such cases, as the infection wanes, the reproduction number *R*_0_ is not constant, but rather increases in an algebraic manner with respect to *i*. The conclusion that the emergence of a power law in time requires that *R*_0_ must increase as the infection wanes, is consistent with the social tendency to relax restrictions (e.g. decrease spatial distancing, increase areal densities, etc.) as the epidemic wanes.

Motivated by the fact that the actual data are best matched using an algebraic dependence and that Luhar *et al.* [9] provide a way to incorporate such behaviour in a simple SIR model, we will extend here this methodology to a more elaborate SEIR-type model, that also includes hospitalized and deceased patients data, neither of which are included in the simpler SIR model of [9].

The paper is organized as follows: in the Formulation and results section, we present a modification of the SEIR model that accurately gives rise to an algebraic asymptotic behaviour. We then fit the experimental results on the epidemiological data for the deceased of the first wave in Portugal to the algebraic and birational formulae (1.2) and (1.3). These formulae together with the new model are then used to solve the inverse problem and to determine the various parameters of our full mechanistic novel model. Note that, in a certain sense, the mathematical expressions for our forecasting models are optimal, since an elaborate deep-learning algorithm does not seem to yield better results. In solving *the inverse problem*, all parameters of the new SEIR-type model are determined from the knowledge of the deceased and hospitalized data. It is shown that the numerical solution matches well the analytical asymptotic results. Furthermore, as elaborated in the Discussion section, this solution provides a confirmation of the accuracy of the solution of the inverse problem.

## 2. Materials and methods

### 2.1. Formulation and results

**Figure 2.**
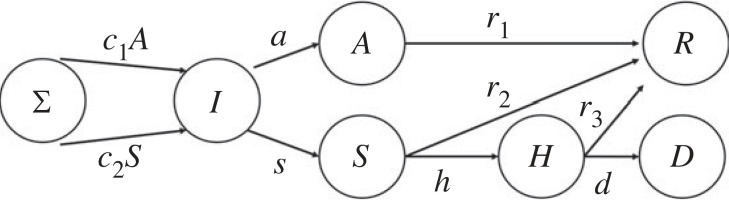
Flow diagram and transmission rates for the different populations in the SEIR model analysed in this work.

The SEIR model previously discussed in several publications [11,12] contains the following populations: Σ(*t*), *I*(*t*), *A*(*t*), *S*(*t*), *H*(*t*), *R*(*t*) and *D*(*t*), which denote susceptible, infected, asymptomatic, sick, hospitalized, recovered and deceased individuals, respectively. In this notation, *I*(*t*) refers to individuals infected, but not yet infectious, since it is generally assumed that infectiousness requires the elapse of a certain incubation period (typically 5 days) [12]. Then, that individual will either become sick or asymptomatic, both of which are infectious. Importantly, this means that a susceptible individual joins the population *I*(*t*) only because of encounters with individuals from populations *A*(*t*) or *S*(*t*) (but not from *I*(*t*)). The various entities are interdependent, as indicated in figure 2: if the daily rates at which an infected person becomes either sick or asymptomatic are *s* or *a*, respectively, then each day *sI*(*t*) or *aI*(*t*) individuals leave population *I*(*t*) and enter *S*(*t*) and *A*(*t*), respectively. Likewise, if asymptomatic individuals recover at a daily rate *r*_1_, each day *r*_1_*A*(*t*) individuals leave the asymptomatic population and enter the recovered population, *R*(*t*). Sick individuals either recover at a rate *r*_2_ or they become hospitalized at a rate *h*. The hospitalized patients also have two possible outcomes: either they recover at a rate *r*_3_, or they are deceased at a rate *d*. Finally, susceptible individuals convert to infected, but not yet infectious, populations, because of their encounter with either asymptomatic or sick individuals (we assume that due to special precautions, hospitalized populations cannot infect) at corresponding rates *c*_1_*A* and *c*_2_*S*, where *c*_1_ and *c*_2_ denote the respective transmission rates (per person in the respective pools). It is important to note that from all these coefficients, it is only *c*_1_ and *c*_2_ that can be largely affected by social dynamics, the other being largely independent.

### 2.2. The basic formulation

The previous description leads to the following seven ordinary differential equations:

2.1 $$A^{\left(1\right)}=aI-r_{1}A,$$

2.2 $$S^{\left(1\right)}=sI-(h+r_{2})S,$$

2.3 $$H^{\left(1\right)}=hS-(r_{3}+d)H,$$

2.4 $$R^{\left(1\right)}=r_{1}A+r_{2}S+r_{3}H,$$

2.5 $$D^{\left(1\right)}=dH,$$

2.6 $$\Sigma^{\left(1\right)}=-\Sigma (c_{1}A+c_{2}S)$$

2.7 $$\mathrm{and}\;\;I^{\left(1\right)}=\Sigma (c_{1}A+c_{2}S)-(a+s)I,$$

where superscript (*n*) indicates *n*th time derivative. Total conservation of individuals leads to

2.8 I+S+A+R+H+D+Σ=T,

where *T* is the sum of all individuals in the control volume prior to the pandemic.

Eliminating Σ from equations (2.6) and (2.7) leads to

2.9 $$I^{\left(1\right)}=[T-(I+S+A+R+H+D)](c_{1}A+c_{2}S)-(A+s)I.$$

We will next proceed with the assumption that the coefficients *a*, *r*_1_, *s*, *h*, *r*_2_, *r*_3_ and *d* are constants. Then, for arbitrary, and not necessarily constant, values of *c*_1_ and *c*_2_, equations (2.1–2.7) can be reduced to a system of two ODEs. The final result reads as follows:

Populations *H*, *S* and *I* can be expressed in terms of *D*, via the following equations:

2.10a $$H=\frac{D^{\left(1\right)}}{d},$$

2.10b $$S=\frac{1}{dh}(D^{\left(2\right)}+R_{3}D^{\left(1\right)})$$

2.10c $$\mathrm{and}\;\;I=\frac{1}{dhs}[D^{\left(3\right)}+(R_{2}+R_{3})D^{\left(2\right)}+R_{2}R_{3}D^{\left(1\right)}].$$

Also, equations (2.1) and (2.2) can be written in the form

2.11 $$\begin{array}{rl} A= & \frac{a}{r_{1}}I-\frac{A^{\left(1\right)}}{r_{1}}\\ \mathrm{and}\;\;S= & \frac{s}{h+r_{2}}I-\frac{S^{\left(1\right)}}{h+r_{2}}. \end{array}\}$$

**Proof of equations (2.10):** Equation (2.5) is equation (2.10*a*). Solving equation (2.3) for *S* and then replacing *H* via (2.10*a*) we find (2.10*b*). Solving (2.2) for *I* and then replacing *S* via (21.10*b*) we find (2.10*c*). Adding (2.1) and (2.4) and then replacing *S*, *H*, *I* via (2.10) we find,

2.12 $$\begin{array}{rl} {\left(A+R\right)}^{\left(1\right)}= & \frac{1}{dhs}[aD^{\left(3\right)}+[a(R_{2}+R_{3})+sr_{2}]D^{\left(2\right)}\\ & \;+[aR_{2}R_{3}+s(hr_{3}+r_{2}R_{3})]D^{\left(1\right)}]. \end{array}$$

Integrating this equation and simplifying, we obtain

2.13 $$A+R=\frac{aD^{\left(2\right)}}{dhs}+[a(R_{2}+R_{3})+sr_{2}]\frac{D^{\left(1\right)}}{dhs}+(FR_{2}R_{3}-dhs)\frac{D}{dhs}+\xi ,$$

where *ξ* is an integration constant. Replacing in (2.1) the function *I* via (2.10*c*), we obtain (2.22).

Now, equation (2.9) is

2.14 $$I^{\left(1\right)}+(a+s)I=[T-(I+S+A+R+H+D)](c_{1}A+c_{2}S).$$

*QED*

Replacing in (2.6) and (2.7) *A* and *S* via (2.11), we find

2.15 $$\begin{array}{rl} \Sigma^{\left(1\right)}= & -\Sigma I\left(\frac{c_{1}a}{r_{1}}+\frac{c_{2}s}{h+r_{2}}\right)+\Sigma \left(\frac{c_{1}A^{\left(1\right)}}{r_{1}}+\frac{c_{2}S^{\left(1\right)}}{h+r_{2}}\right)\\ I^{\left(1\right)}= & -(a+s)I\left[1-\frac{1}{a+s}\left(\frac{c_{1}a}{r_{1}}+\frac{c_{2}s}{hr_{2}}\right)\Sigma \right]-\Sigma \left(\frac{c_{1}A^{\left(1\right)}}{r_{1}}+\frac{c_{2}S^{\left(1\right)}}{h+r_{2}}\right), \end{array}\}\;$$

where *R*_2_ and *R*_3_ are defined by

2.16 $$R_{2}=r_{2}+h,\;R_{3}=r_{3}+d.$$

In order to make use of the results in Luhar *et al.* [9], we next consider the hypothetical case, *A*^(1)^ = *S*^(1)^ = 0, in which case equations (2.15) become

2.17 $$\begin{array}{rl} I^{\left(1\right)}= & -(a+s)I\left[1-\frac{1}{a+s}\left(\frac{c_{1}a}{r_{1}}+\frac{c_{2}s}{h+r_{2}}\right)\Sigma \right]\\ \mathrm{and}\;\;\;\;\;\;\Sigma^{\left(1\right)}= & -\Sigma I\left(\frac{c_{1}a}{r_{1}}+\frac{c_{2}s}{h+r_{2}}\right). \end{array}\}$$

These are in a form comparable to the SIR model previously described [9], written here as

2.18 $$\begin{array}{rl} \frac{dI}{d\tau }= & -I\left(1-\frac{Rs}{1+x^{1/n}}\right),\;x={\left(\lambda -1\right)}^{n}\frac{I}{I_{1}}\\ \mathrm{and}\;\;\;\;\;\;\frac{d\Sigma }{d\tau }= & -I\Sigma \frac{R}{1+x^{1/n}}. \end{array}\}$$

The two sets of equations (2.17, 2.18) become self-consistent if we take

τ=(a+s)t,

and

2.19 $$\frac{1}{a+s}\left(\frac{c_{1}a}{r_{1}}+\frac{c_{2}s}{h+r_{2}}\right)=\frac{R}{1+x^{1/n}}.$$

Thus, consistency implies the following dependence for the variables *C*_1_ and *C*_2:_

2.20 $$\begin{array}{rl} C_{1}(I)\equiv & \frac{ac_{1}}{dhs}=\frac{\rho_{1}}{1+\Lambda_{1}I^{1/n}}\\ \mathrm{and}\;\;\;\;\;\;\;\;C_{2}(I)\equiv & \frac{c_{2}}{dh}=\frac{\rho_{2}}{1+\Lambda_{2}I^{1/n}}, \end{array}$$

where *ρ*_j_, Λ*_j_*, *j* = 1,2, are constants. The subsequent analysis will assume that most of the infection process is via exposure to asymptotic individuals, hence we will take *C*_1_(*I*) >> *C*_2_(*I*).

In order to proceed, we will make use of the following results. Let $\tilde{A}$ and *Q* be defined in terms of *A* and *D* via

2.21a $$\tilde{A}=\frac{dhs}{a}A$$

and

2.21b $$Q=D^{\left(3\right)}+(F+R_{2}+R_{3})D^{\left(2\right)}+(FR_{2}+FR_{3}+R_{2}R_{3})D^{\left(1\right)}+FR_{2}R_{3}D-\alpha ,$$

Then, the functions $\tilde{A}$ and *D* satisfy the two ODEs,

2.22 $${\tilde{A}}^{\left(1\right)}+r_{1}\tilde{A}=D^{\left(3\right)}+(R_{2}+R_{3})D^{\left(2\right)}+R_{2}R_{3}D^{\left(1\right)}$$

and

2.23 $$\frac{Q^{\left(1\right)}}{Q}+C_{1}\tilde{A}+C_{2}(D^{\left(2\right)}+R_{3}D^{\left(1\right)})=0,$$

where

F=a+s

2.24 α=dhs(T−ξ),

with *ξ* a constant of integration.

**Proof of equations (2.22) and (2.23):** It is straightforward to verify that the l.h.s. of (2.14) equals *Q*^(1)^/d*hs*, with *Q* defined in (2.21b). Furthermore, remarkably, equations (2.13) and (2.10) imply that the bracket of the r.h.s. of (2.14) is − *Q*/d*hs*. Then, (2.14) becomes (2.23). QED

### 2.3. Large time asymptotic analysis

With $\tilde{A}$ and *D* satisfying equations (2.22) and (2.23), *C*_1_ given by (2.20) and taking *C*_2_ < <*C*_1_, one can show that *D* approaches asymptotically the constant value *D*_∞_, and the dependence of *σ* on *t* for large *t* is not exponential

2.25 $$D\to D_{\infty }(1+\sigma ),t\to \infty ,\sigma \ll 1\;\sigma^{\left(\;j+1\right)}\ll \sigma^{\left(j\right)},\;j=0,1,2,\ldots .$$

The leading behaviour is given by the following equations:

2.26 $$\begin{array}{rl} \sigma ∼ & -\frac{{\left(n-1\right)}^{-1/(n-1)}}{R_{2}R_{3}D_{\infty }}{\left(\frac{\mu }{{\tilde{\Lambda }}_{1}}\right)}^{-n/(n-1)}t^{-1/(n-1)},\;\mu =\frac{\rho_{1}}{r_{1}},\\ I∼ & \frac{{\left(n-1\right)}^{-n/(n-1)}}{dhs}{\left(\frac{\mu }{{\tilde{\Lambda }}_{1}}\right)}^{-n/(n-1)}t^{-n/(n-1)}\\ \mathrm{and}\;\;\;\;\;\;\tilde{A}∼ & \frac{{\left(n-1\right)}^{-n/(n-1)}}{r_{1}}{\left(\frac{\mu }{{\tilde{\Lambda }}_{1}}\right)}^{-n/(n-1)}t^{-n/(n-1)},\;t\to \infty . \end{array}\}$$

**Proof of equations (2.26):** Substituting (2.25) in equation (2.22), in the definition of *Q* (equation (2.21*b*)), and in equation (2.10*c*), we find the following equations:

2.27a $${\tilde{A}}^{\left(1\right)}+r_{1}\tilde{A}∼{\tilde{\sigma }}^{\left(1\right)},$$

2.27b $$\mathrm{with}\;\tilde{\sigma }=R_{2}R_{3}D_{\infty }\sigma ,$$

2.28 $$Q∼F(v+\tilde{\sigma }),\;v=R_{2}R_{3}D_{\infty }-\frac{\alpha }{F},$$

2.29 $$\mathrm{and}\;I∼\frac{{\tilde{\sigma }}^{\left(1\right)}}{dhs},\;t\to \infty$$

It turns out that equation (2.27*a*) implies

2.30 $$\tilde{A}∼\frac{{\tilde{\sigma }}^{\left(1\right)}}{r_{1}},\;t\to \infty .$$

Indeed, integrating (2.27*a*) we find

2.31 $$\tilde{A}e^{r_{1}t}=\mathrm{constant}+\int_{\;}^{t}e^{r_{1}\tau }{\tilde{\sigma }}^{\left(1\right)}(\tau )d\tau .$$

Integration by parts implies

$$\int_{\;}^{t}e^{r_{1}\tau }{\tilde{\sigma }}^{\left(1\right)}(\tau )\;d\tau =\frac{e^{r_{1}t}{\tilde{\sigma }}^{\left(1\right)}(t)}{r_{1}}+\mathrm{const}-\frac{1}{r_{1}}\int_{\;}^{t}e^{r_{1}\tau }{\tilde{\sigma }}^{\left(2\right)}(\tau )\;d\tau .$$

Substituting this expression in (2.31) and then multiplying by $e^{-r_{1}t}$, we find (2.30).

Letting in equation (2.23) *C*_2_ = 0 and then using the expressions for *Q*, *I*, $\tilde{A}$ from (2.28), (2.29) and (2.30), we find

$$\sigma^{\left(1\right)}\left[1+\frac{v+\tilde{\sigma }}{r_{1}}C_{1}\right]∼0,\;t\to \infty .$$

Using in this equation, the expression of *C*_1_ from (2.20) we obtain

$$1+\frac{(v+\tilde{\sigma })\mu }{1+{\tilde{\Lambda }}_{1}{\left(\sigma^{\left(1\right)}\right)}^{1/n_{1}}}=0,\;t\to \infty$$

and

2.32 $$1+v\mu +{\tilde{\Lambda }}_{1}{\left({\tilde{\sigma }}^{\left(1\right)}\right)}^{1/n_{1}}+\mu \tilde{\sigma }=0.$$

Hence, the requirement that *σ* decays implies

2.33 $$v\mu +1=0,\;{\tilde{\sigma }}^{{\left(1\right)}^{1/n_{1}}}=-\frac{\mu }{{\tilde{\Lambda }}_{1}}\tilde{\sigma }.$$

Integrating (2.33*b*), we find

2.34 $$\tilde{\sigma }=-{\left(n_{1}-1\right)}^{-\frac{1}{n_{1}-1}}{\left(\frac{\mu }{{\tilde{\Lambda }}_{1}}\right)}^{-n_{1}/(n_{1}-1)}t^{-1/(n_{1}-1)},\;t\to \infty .$$

Then, (2.27*b*), (2.29) and (2.30) yield (2.26).

*QED*

In the subsequent section, we will fit the full equations to the experimental data with goal to determine the various parameters.

We proceed with the assumption *C*_2_ = 0. First, we will consider fitting the data to the algebraic birational formula in (1.3).

### 2.4. Bidirectional long short-term memory network

Bidirectional long short-term memory (BiLSTM) is a powerful generalization of recurrent neural networks that can capture long-term dependencies while at the same time avoiding the problem of vanishing/exploding gradients. The BiLSTM networks are well suited for time series prediction and can potentially completely capture the contextual information of the time series. The BiLSTM network employed in this work was introduced to predict the number of infected for different countries in [13].

## 3. Results

### 3.1. An algebraic formula and deep learning

We used data for the cumulative number of deceased, *D*(*t*), and the total number of hospitalized patients, *H^c^*(*t*), from the first wave of COVID-19 in Portugal dating from March to September 2020. During this period, Portugal experienced relatively low numbers of infected, although a larger number of deaths compared with other countries with similar populations, such as Greece. The corresponding data were fitted using the two formulae

3.1 $$D(t)=\frac{D_{F}}{1+\beta {\left(1+\delta t\right)}^{-\gamma }},\;H^{c}(t)=\frac{H_{F}^{c}}{1+\tilde{\beta }{\left(1+\tilde{\delta }t\right)}^{-\tilde{\gamma }}},$$

as well as the analogous formulae for the birational model.

It can be shown that the following useful formula is valid:

3.2 $$H^{c}=H+D+R_{H},$$

and *R_H_* denotes the number of people that recovered after they were hospitalized. We used *H^c^*, as this was the more accurate data reported. The respective parameters, shown in table 1, were determined using data up to the middle of August and the simplex optimization algorithm.

**Table 1.** Estimated parameters of equation (3.1) when fitted to the number of deceased, *D*(*t*), and the total number of hospitalized patients, *H^c^*(*t*), for the first wave in Portugal. The confidence intervals for each fitting parameter are also presented.

		deceased	hospitalized
logistic	*D_F_*	1626.5 (1576.2,1669.93)	4.436 × 10^+04^ (4.297 × 10^4^, 4.552 × 10^4^)
	*β*	5.429 (4.962, 5.867)	47.456 (45.106, 48.711)
	*γ*	0.056 (0.05, 0.061)	0.045 (0.040, 0.049)
	R2	0.9836 (0.976, 0.989)	0.978 (0.970, 0.986)
rational	*D_F_*	1995.7 (1894.3,2077.3)	5.326 × 10^4^ (5.145 × 10^4^, 5.463 × 10^4^)
	*β*	41.42 (37.33,44.12)	1.969 × 10^4^ (1.901 × 10^4^, 1.198 × 10^4^)
	*δ*	0.298 (0.273,0.319)	0.339 (0.322, 0.353)
	*γ*	1.52 (1.404, 1.614)	2.845 (2.713, 2.926)
	R2	0.988 (0.983, 0.993)	0.987 (0.982, 0.992)
birational	*D_F_*__1__	2973.9 (2824.8, 3095.6)	9.390 × 10 × 10^+04^ (9.076 × 10^4^, 9.64 × 10^4^)
	*β*_1_	39.713 (37.105, 41.62)	760.037 (728.04, 781.36)
	*δ*_1_	0.175 (0.156, 0.189)	0.029 (0.020, 0.038)
	*γ*_1_	1.562 (1.476, 1.664)	4.554 (4.067, 4.890)
	T	30	81
	*D_F_*__2__	1912.5 (1815.5, 1990.1)	2.285 × 10^+04^ (2.201 × 10^4^, 2.343 × 10^4^)
	*β*_2_	57.307 (54.4, 60.21)	1.559 × 10^+04^ (1.509 × 10^4^, 1.611 × 10^4^)
	*δ*_2_	0.3796 (0.36, 0.41)	0.0150 (0.009, 0.020)
	*γ*_2_	1.410 (1.33, 1.467)	9.286 (8.167, 9.971)
	R2	0.999 (0.998, 1)	0.990 (0.986, 0.994)
BiLSTM	R2	0.999 (0.998, 1)	0.998 (0.997, 0.999)

**Figure 3.**
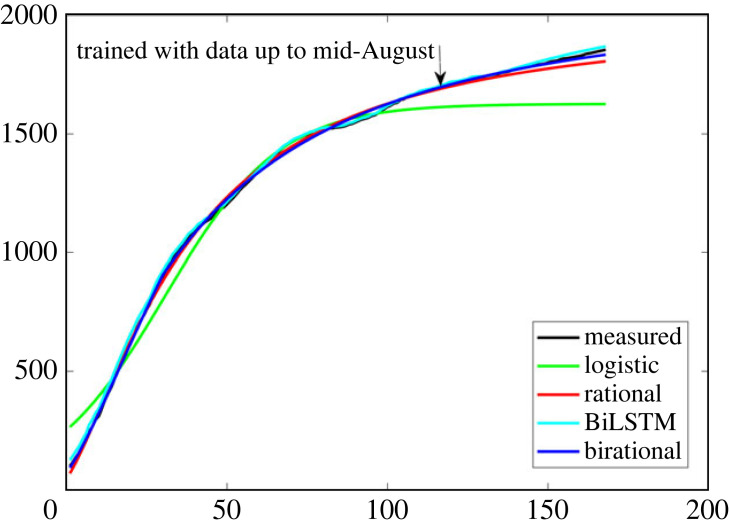
Comparison of the BiLSTM, the logistic, the rational and the birational models for the number of deceased.

We note that the above formulae provide an optimal fit, given that the use of the efficient deep learning algorithm, BiLSTM network [14], provided similar results. Figure 3 shows the performance of BiLSTM against the logistic and rational models for the number of diseased and hospitalized. Both BiLSTM and the rational had a similar strong correlation to the measured data (*R*^2^ ~ 0.999), with the logistic expression resulting into a smaller value *R*^2^ ~ 0.98.

### 3.2. The solution of the inverse problem

We solved the inverse problem by employing our new mechanistic model together with both the rational and the birational formulae.

We used the fact that

3.3 $$\begin{array}{rl} R_{H}^{\left(1\right)}= & r_{3}H,\\ H= & \frac{D^{\left(1\right)}}{d}. \end{array}$$

Hence,

3.4 $$R_{H}=\frac{r_{3}}{d}D+g.$$

Equation (3.2) became equation (3.5), where we used *R*_3_ = *r*_3_ + *d*.

3.5 $$H^{c}=\frac{1}{d}(D^{\left(1\right)}+R_{3}D)+g,\;g\;\mathrm{constant}.$$

We used equation (3.1) to match (3.5), and thus, we obtained with the aid of the simplex algorithm, the values for parameters *R*_3_, *d* and *g*, as shown in table 2.

**Table 2.** Determination of the constant parameters in equation (3.5) and their respective confidence intervals.

	rational	birational
*R*_3_	0.061 (0.055, 0.066)	0.0645 (0.0580, 0.0707)
*d*	9.232 × 10^−04^ (8.583 × 10^−04^, 1.106 × 10^−03^)	0.0010 (8.655 × 10^−04^, 1.158 × 10^−03^)
*g*	−76172 (−72860, −79483)	−77020 (−71932, 80785)

To determine the remaining parameters, we first introduce the notation

3.6 $$X=D^{\left(2\right)}+(R_{2}+R_{3})D^{\left(1\right)}+R_{2}R_{3}D.$$

Equations (2.22) and (2.23) read,

$${\tilde{A}}^{\left(1\right)}+r_{1}\tilde{A}=X^{\left(1\right)},$$

$$\frac{X^{\left(2\right)}+FX^{\left(1\right)}}{X^{\left(1\right)}+FX-a}=-C_{1}\tilde{A}$$

and

3.7 $$C_{1}=\frac{\rho_{1}}{1+{\tilde{\Lambda }}_{1}X^{1/n}}.$$

Equations (3.7), with the aid of the simplex algorithm, yield the values of the remaining constant parameters shown in table 3.

**Table 3.** Determination of the constant parameters in equation (3.7) and their respective confidence intervals.

	rational	birational
*F*	0.989 (0.955, 1.022)	0.992 (0.946, 1.036)
*R*_2_	0.116 (0.097, 0.134)	0.131 (0.108, 0.147)
*a*	5716.6 (5344.4, 6056)	5352.7 (4932.5, 5833.4)
*r*_1_	0.798 (0.701, 0.898)	0.861 (0.732, 0.977)
*ρ*_1_	1.404 (1.22 × 10^−04^, 1.57 × 10^−04^)	1.520–04 (1.370 × 10^−04^, 1.64 × 10^−04^)
${\tilde{\Lambda }}_{1}$	0.131 (0.123, 0.139)	0.133 (0.124, 0.144)
*n*	4.002 (3.768, 4.385)	4.026 (3.734, 4.436)

As expected, the value for *n* satisfies the constraint *n* > 2.

### 3.3. Numerical verification

Finally, we verified that the numerical solution of our mechanistic model matches, for large *t*, the rational formula. Similar considerations are valid for the birational model. For this purpose, we used the values of the various constants (tables 2 and 3), obtained from the experimental data. In this connection, it is interesting to note that from the whole set of equations (2.1)–(2.7), we only need to solve the following smaller set of four differential equations for the variables $N,Q,\;\tilde{A},\;M$:

3.8a $$N^{\left(1\right)}=Q+\alpha -FN,$$

3.8b $$\frac{Q^{\left(1\right)}}{Q}=-C_{1}\tilde{A}-C_{2}M,$$

3.8c $${\tilde{A}}^{\left(1\right)}=-r_{1}\tilde{A}+Q+\alpha -FN$$

3.8d $$\mathrm{and}\;\;\;\;\;\;\;\;M^{\left(1\right)}=Q+\alpha -FN-R_{2}M,$$

where

$$M(t)=R_{3}D^{\left(1\right)}(t)+D^{\left(2\right)}(t)$$

and

3.9 $$N(t)=R_{2}(R_{3}D(t)+D^{\left(1\right)}(t))+R_{3}D^{\left(1\right)}(t)+D^{\left(2\right)}(t).$$

We will take *C*_2_ = 0 and $C_{1}=\rho_{1}/1+{\tilde{\Lambda }}_{1}{\left(Q+\alpha -FN\right)}^{1/n}$.

**Proof of equations (3.8):** The definitions of *Q* and *N* imply the identity

3.10 $$D^{\left(3\right)}+(R_{2}+R_{3})D^{\left(2\right)}+R_{2}R_{3}D^{\left(1\right)}=Q+\alpha -FN,$$

which is equation (3.8*a*). Also, using the definition of *M*, equation (2.23) becomes (3.8*b*). Similarly, using (3.10), equation (2.22) becomes (3.8*c*). Equation (3.8*d*) follows from the fact that the l.h.s. of (3.10) can be written in the form *M*^(1)^ + *R*_2_*M*.

*QED*

**Definition of equations (3.8) at some fixed *t*:** The definition of $N,Q,\;\tilde{A},\;M$ of equations (3.8) at some fixed *t*, denoted by *t*_0_, imply the following formulae:

$$N(t_{0})=R_{2}(R_{3}D(t_{0})+D^{\left(1\right)}(t_{0}))+R_{3}D^{\left(1\right)}(t_{0})+D^{\left(2\right)}(t_{0}),$$

$$Q(t_{0})=D^{\left(3\right)}(t_{0})+(R_{2}+R_{3}+F)D^{\left(2\right)}(t_{0})+(FR_{2}+FR_{3}+R_{2}R_{3})D^{\left(1\right)}(t_{0})+FR_{2}R_{3}D(t_{0})-\alpha$$

3.11 $$M(t_{0})=R_{3}D^{\left(1\right)}(t_{0})+D^{\left(2\right)}(t_{0}).$$

Also,

3.12 $$\tilde{A}(t_{0})=-\frac{1}{C_{1}(t_{0})}\frac{Q^{\left(1\right)}(t_{0})}{Q(t_{0})},$$

where

3.13 $$C_{1}(t_{0})=\frac{\rho_{1}}{1+{\tilde{\Lambda }}_{1}{\left[D^{\left(3\right)}(t_{0})+(R_{2}+R_{3})D^{\left(2\right)}(t_{0})+(R_{2}R_{3})D^{\left(1\right)}(t_{0})\right]}^{1/n_{1}}}.$$

Under the assumption that *C*_2_ = 0 and having defined $N,Q,\;\tilde{A},\;M$ at some fixed *t*_0_, we solved equations (3.8) numerically using the Runge–Kutta method and the following expression for *D*(*t*),

3.14 $$D(t)=\frac{1995.7}{1+41.42{\left(1+0.298t\right)}^{-1.52}}.$$

**Figure 4.**
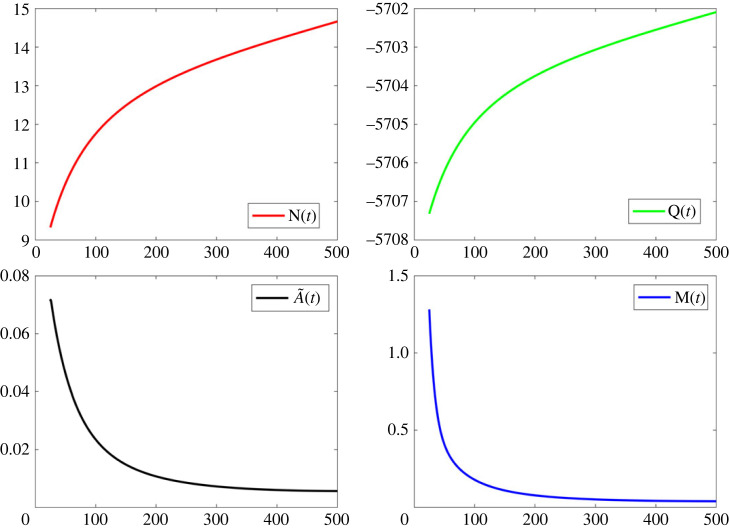
Illustration of the numerical solutions for the functions *M*(*t*), *Q*(*t*), $\tilde{A}(t)$ and *N*(*t*).

Results are shown in figure 4.

The function *σ* can be determined via

3.15 $$D^{\left(1\right)}(t)+R_{3}D(t)=\frac{N(t)-M(t)}{R_{2}},$$

which yields

3.16 $$D_{\infty }(\sigma^{\left(1\right)}(t)+R_{3}\sigma (t))=\frac{N(t)-M(t)}{R_{2}}-R_{3}D_{\infty }.$$

**Figure 5.**
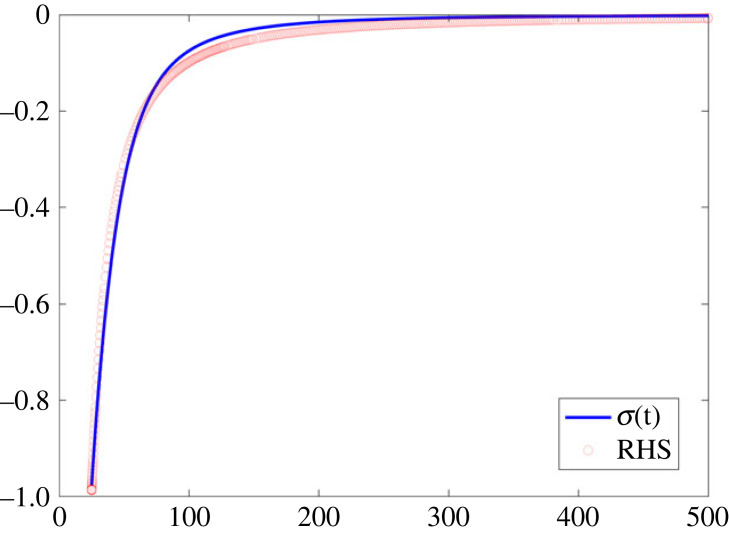
Illustration of the function *σ*(t) and of the r.h.s. of equation (3.17).

The solution of this equation with *σ*(*t*_0_) = *D*(*t*_0_)/*D*_∞_ − 1 is depicted in blue in figure 5. By plotting in the same figure, the equation depicted in red, we find

3.17 $$\sigma ∼-\frac{\mathrm{constant}}{t^{m}},\;t\to \infty .$$

It becomes clear that for large *t*, the function *σ*(*t*) approaches the r.h.s. of (3.17).

The first of equations (2.33) implies the constraint *vμ* + 1 = 0. Using in this equation the expressions for *v* and *μ* given in (2.26) and (2.28) we find the equation

3.18 $$\left(R_{2}R_{3}D_{\infty }-\frac{\alpha }{F}\right)\left(\frac{\rho_{1}}{r_{1}}\right)+1=0.$$

Employing the values obtained via the solution of the inverse problem, it can be verified that equation (3.18) is indeed approximately satisfied: using for the constants appearing in the r.h.s. of (3.18) and the numerical values of the constants obtained in tables 2 and 3, we find that the r.h.s. of (3.18) is −0.014 (instead of zero).

## 4. Discussion

The forecasting models [2,3] suggest that the dynamics of the first wave of COVID-19 approach an equilibrium state algebraically, as opposed to the exponential behaviour normally predicted by the standard SEIR mechanistic models. Motivated by these results, we introduced a novel mechanistic model which for large times does exhibit algebraic behaviour. This model takes into consideration an additional nonlinear mechanism first understood in the context of SIR models in [9,10]. Specifically, in comparison with the standard SEIR models, such as those previously analysed in [11,12], we allowed for the values of the transmission constants to increase as the infection wanes, which is reflecting the social behavioural tendency to be less cautious as the infection diminishes. The specific relationship used fits well the actual data. As a result of this modification, the asymptotic analysis of the equations defining the new model yields the algebraic behaviour predicted by equations (3.8).

It should be emphasized that several authors have previously published papers addressing the need to incorporate sub-exponential/power law dynamics. Some of these papers are mentioned below. The main novelty of our SEIR-type model, in comparison with the earlier very interesting papers, is the introduction of an epidemiologically motivated mechanistic model which allows to determine the associated large *t*-dynamics. Indeed, as we hope will become clear in what follows, the previous models concentrated on the early times behaviour. In particular, Chowell *et al.* [15], addressed early reactive behaviour changes associated with the 2014–2015 Ebola epidemic in West Africa, which exhibited a slower than exponential growth pattern. This was modelled by the equation

4.1 $$\frac{dc}{dt}=rc^{p}\;\;\;\;\;\;\;\;0\leq p\leq 1,$$

which can also be used to model the spread of a range of pathogens, including the epidemics of influenza, Ebola, foot-and-mouth disease, HIV/AIDS, plague, measles and smallpox. Equation (4.1) was also investigated by Viboud *et al.* [16].

An extended version of (4.1), namely, the equation

4.2 $$\frac{dc}{dt}=rc^{p}\left(1-{\frac{C}{k}}^{a}\right)\;\;\;0\leq p\leq 1\;$$

was investigated in [17].

Several modifications of the standard SIR epidemic model to support early sub-exponential growth dynamics are discussed by Chowell *et al.* [15], including the following: (i) metapopulation models, namely characterizing populations in terms of subpopulations (e.g. based on age, vulnerability, etc.), (ii) incorporating reactive behaviour changes by modelling a time-dependent transmission rate, and (iii) incorporating inhomogeneous mixing through a power law scaling parameter. These considerations motivated the introduction of the SIR type model,

4.3 $$\begin{array}{rl} \frac{dS}{dt}= & \frac{-\beta (t)SI}{N},\\ \frac{dI}{dt}= & \frac{\beta (t)SI}{N}-\gamma I\\ \mathrm{and}\;\;\frac{dR}{dt}= & \gamma I. \end{array}\}$$

This model can be simply interpreted as an SIR model with a time-varying reproduction number.

The models mentioned above were used in the paper by Chowell *et al.* [18] in order to fit the early epidemiological data in a number of infections.

Another important contribution of our work is that it addresses a well-known difficulty associated with mechanistic models, namely the determination of the values of the various parameters that enter in the models. In this paper, we present a computationally efficient and robust approach for solving this inverse problem. Moreover, we show that knowledge of the numbers of deceased and hospitalized patients is sufficient to determine all the parameters of the model uniquely and accurately.

The numerical solution presented is useful for two reasons. First, the numerical results confirmed the validity of the asymptotic analysis. Indeed, the numerical solution matched well the asymptotic formula, *σ* ∼ *t*^−*m*^. Second, they confirmed the validity of the solution of the inverse problem. Indeed, the asymptotic analysis gives rise to the constraint *νμ* + 1 = 0. Since *ν* and *μ* can be expressed in terms of the model parameters determined via the solution of the inverse problem, this equation provides a check of the accuracy of the solution of the inverse problem.

Concluding, it is worth noting that in addition to SEIR models several other types of models have been introduced in connection with COVID-19 [19–22]. It would be interesting to investigate whether any of these models exhibit large *t* algebraic behaviour.

## Data Availability

Data and relevant code for this research work are stored in GitHub: https://github.com/dssg-pt/covid19pt-data and have been archived within the Zenodo repository: https://zenodo.org/record/8131537.
